# Influence of Superhydrophobic Coating on the Water Resistance of Foundry Dust/Magnesium Oxychloride Cement Composite

**DOI:** 10.3390/ma13153431

**Published:** 2020-08-04

**Authors:** Fajun Wang, Xiantao Zhu, Huangjuan Liu, Sheng Lei, Daqi Huang

**Affiliations:** 1 School of Materials Engineering, Jiangsu University of Technology, Changzhou 213001, China; jjbxsjz@foxmail.com (F.W.); memory199251@foxmail.com (X.Z.); lhj0313@foxmail.com (H.L.); 2 Shiyan Litong New Materials Technology Co., LTD, Shiyan 442599, China; liu-corr@foxmail.com

**Keywords:** foundry dust, waste recycling, magnesium oxychloride cement, superhydrophobic coating, water resistance

## Abstract

In this work, magnesium oxychloride cement (MOC) was used to realize the resource use of foundry dust (FD). Portland cement (PC)-based superhydrophobic coating was prepared on the surface of FD/MOC composite to improve the water resistance of the composite. First, the FD/MOC composites with different contents of FD were prepared. The phase structure of the composite was analyzed using X-ray diffraction (XRD). The microstructure of the cross-section and surface of the composite was observed using field emission scanning electron microscope (FE-SEM). The mechanical properties of the FD/MOC composites with different FD contents at different ages were tested and analyzed. Secondly, the superhydrophobic coating was prepared on the surface of MOC composite using silane/siloxane aqueous emulsion as the hydrophobic modifier, PC as the matrix and water as the solvent. The microstructure and chemical composition of the PC-based superhydrophobic coating were tested and analyzed. The results show that FD can significantly improve the early strength of the FD/MOC composite. The 28-day compressive strength of the FD/MOC composite decreases with increasing FD content. When the FD content is 30%, the 28-day compressive strength of the FD/MOC composite is as high as 75.68 MPa. Superhydrophobic coating can effectively improve the water resistance of the FD/MOC composite. The softening coefficient of the FD/MOC composite without superhydrophobic coating is less than 0.26, while that of the composite modified by superhydrophobic coating is greater than 0.81.

## 1. Introduction

Magnesium oxychloride cement (MOC) was invented by French scientist Sorell and belongs to the category of special cement [1,2,3]. The main hydration products of MOC at room temperature are Phase 5 [5Mg(OH)_2_·MgCl_2_·8H_2_O] and Phase 3 [3Mg(OH)_2_·MgCl_2_·8H_2_O] [3]. The main raw materials for producing MOC are light burned magnesium oxide and halogen tablets [MgCl_2_·6H_2_O]. Compared with Portland cement (PC), MOC is a kind of air hardening gel material. MOC has the advantages of high early strength, high strength, fire resistance, corrosion resistance, and wear resistance.

However, the water resistance of MOC is very poor [1,2,3]. The strength of MOC decreases sharply after continuous exposure to water, which seriously affects its use. Different methods have been proposed to improve the water resistance of MOC, among which the addition of supplementary cementitious materials or additives is the most effective method [4,5,6]. It has been reported that the water resistance of MOC can be significantly improved by introducing a small amount of phosphoric acid, phosphate, fly ash, rice husk ash, or composite modifier into the MOC [2,7,8,9]. However, these additives usually reduce the strength of MOC, which is not conducive to the resource use of industrial solid waste.

Industrial production produces a lot of solid waste, such as foundry waste, waste paper sludge, municipal waste incineration fly ash, etc. The amount of solid waste is very huge, and it is in urgent need for rational disposal and use. The most direct and simplest method is to add these wastes to cement to produce cement composites, which can greatly reduce the cost of cement and use a large amount of solid wastes. PC is the most used cementitious material [10,11,12]. Unfortunately, without special treatment, the hydration activity of these wastes is very low. They can only play a physical filling role in cement. Therefore, when the waste is introduced into the cement, the strength of the cement composite material is significantly reduced. Compared with Portland cement, MOC has obvious advantages in recycling waste. MOC has extremely high strength and can easily reach over 100 MPa [13,14,15]. Therefore, MOC can bind more solid waste while maintaining the same mechanical strength. However, the MOC combined with the waste also needs to face the problem of insufficient water resistance. In this case, the method of adding water-resistant additives will no longer be applicable. This is because the strength of MOC has been reduced when introducing waste into MOC [2,7,8,9]. The strength of the waste/MOC composite will be further reduced when the water-resistant modifier is added to the waste/MOC composite. When the strength of MOC is excessively reduced, it will definitely be detrimental to its application in practical fields. Therefore, when MOC is combined with waste, it is necessary to use other methods which have no adverse effect on its strength to improve its water resistance.

Superhydrophobic coating may be an effective method to improve the water resistance of MOC [16]. The surface contact angle (CA) of the superhydrophobic material is greater than 150° and the sliding angle (SA) is less than 10° [17,18,19]. When a superhydrophobic material is in contact with water, a trapped air layer exists in the interface layer between solid and liquid to separate the solid and liquid phases. Therefore, the actual contact area between superhydrophobic materials and water is very small [20]. These materials have excellent hydrophobicity and can effectively protect materials from water erosion. Silane/siloxane surface impregnant can effectively improve the impermeability of PC-based materials and reduce its water absorption [21,22,23]. However, because the hydration products of MOC and PC are completely different, silane/siloxane modifier is not applicable in the field of MOC. In our preliminary experiment, we found that the silane/siloxane-based surface modification method was ineffective for MOC. Very recently, Qu et al. prepared fluoroalkyl silane (FAS)-based superhydrophobic coating on MOC surface. However, the water resistance of the FAS-based superhydrophobic coating was not investigated [3].

In this work, a compromise method was proposed to solve this problem. First, MOC is used to recycle foundry dust (FD). The traditional method is to use PC to recycle FD. However, due to the low hydration activity, the strength of FD/PC composite is low, which makes the addition amount of FD in the PC is limited. However, the strength of MOC is very high, and high strength FD/MOC composite can be obtained. Therefore, using MOC to recycle FD is an attractive method. Secondly, a superhydrophobic coating based on PC was introduced on the surface of MOC composite because the silane/siloxane-based modifier can endows PC with superhydrophobicity easily [19]. The effect of FD content on mechanical strength of MOC and the effect of superhydrophobic PC coating on water resistance of FD/MOC composite were studied. To the best of our knowledge, no studies have reported on the use of superhydrophobic coatings to improve the water resistance of MOC.

## 2. Materials and Method

### 2.1. Materials

Light calcined magnesia (MgO, the content of MgO is 85% and the activity is 65%) was purchased from Liaoning Yingkou Huiteng Refractory Materials Co. LTD (Yingkou, China). Magnesium chloride hexahydrate (MgCl_2_·6H_2_O, 95%) was purchased from Shandong Yousuo Chemical Technology Co. LTD (Dongying, China). FD was provided by Foundry No.2 Factory of Dongfeng Automobile Co., Ltd. (Hubei, China) and used as received. Water-based hydrophobic emulsion (the active ingredients are silane and siloxane) was purchased from Nanchang Tianyu New Material Technology Co., Ltd. (Nanchang, China). PC (P.O 42.5) was purchased from Shandong Shanshui Cement Group Co. LTD (Dezhou, China).

### 2.2. Preparation

#### 2.2.1. Preparation of FD/MOC Composite

FD/MOC composites with different FD content were prepared according to the formulation depicted in Table 1. In a typical process, magnesium chloride hexahydrate is dissolved in tap water to obtain a transparent solution. Subsequently, MgO powders was added into the solution under mechanical agitation to obtain a MOC paste. Then, the required amount of FD was added into the paste under continuous mechanical agitation. The mixing process lasted ten minutes. The well-mixed FD/MOC composite paste was casted into cubic molds with size of 40 mm × 40 mm × 40 mm. The solidified FD/MOC composite was removed from the mold after overnight. The FD/MOC composites were further cured at ambient condition to different ages before testing and characterization. The FD/MOC composites containing different amount of FD were abbreviated as MOC-xFD, where x is the replacement percentage of dust relative to MgO.

#### 2.2.2. Preparation of Cement Based Superhydrophobic Coating

10.0 g PC and 5.0 g tap water were mixed in a ceramic mortar by hand for 5 min. Then, 0.1 g hydrophobic emulsion was added into the above paste. The cement paste was further mixed for 5 min before coating. The paste was brushed on each surface of the FD/MOC composites to form a coating. The PC coating was further cured at ambient condition for 24 h before characterization.

### 2.3. Characterization

#### 2.3.1. Measurement of Compressive Strength

The compressive strength of the composite was measured by a compressive strength tester. The samples were stored at room temperature for appropriate age, and then the compressive strength was tested. The sample is a cube with a size of 40 mm × 40 mm × 40 mm. Five samples were tested each time and the average value was taken. The compressive strength test is conducted with reference to Chinese standard [JG/T 1169-2005].

#### 2.3.2. Measurement of Softening Coefficient

The softening coefficient test is conducted with reference to standard [ISO 679:1989]. The FD/MOC containing different FD contents were stored at room temperature for 28 days, and the softening coefficient was tested according to the following equation:

*I* = *R*_1_/*R*_0_ (1)

where I is the softening coefficient; R_0_ is the compressive strength of the FD/MOC composite before soaking in water; R_1_ is the compressive strength of the FD/MOC composite after soaking in water for 7 days.

#### 2.3.3. Measurement of Surface Wettability

The CA and SA of the sample surface were measured by optical goniometer (Krüss, DSA30, Hamburg, Germany). 8 µl of deionized water was used as the test liquid. The CA was measured at five different places on the surface of the sample, and then the average value was taken. For SA measurement, the sample is measured on a rotary table that can rotate automatically, and the average value of the five measurements is taken as the measurement result.

#### 2.3.4. XRD, FE-SEM, XPS, and Particle Size Measurement

An x-ray diffractometer (XRD, XRD-7000, Shimadu, Japan) was used to measure the phase structure of the sample; a field emission scanning electron microscope (FE-SEM, QUANTA F150, FEI, Massachusetts, Waltham, America) was used to measure the surface microstructure of the sample; an x-ray photoelectron spectrometer (XPS, Escalab250Xi, Massachusetts, Waltham, America) was used to measure the chemical composition of the coating surface. The particle size distribution of powder was measured by laser particle size analyzer (Marlvern 3000).

## 3. Results and Discussion

### 3.1. MgO and FD Powders Analysis

**Figure 1:**
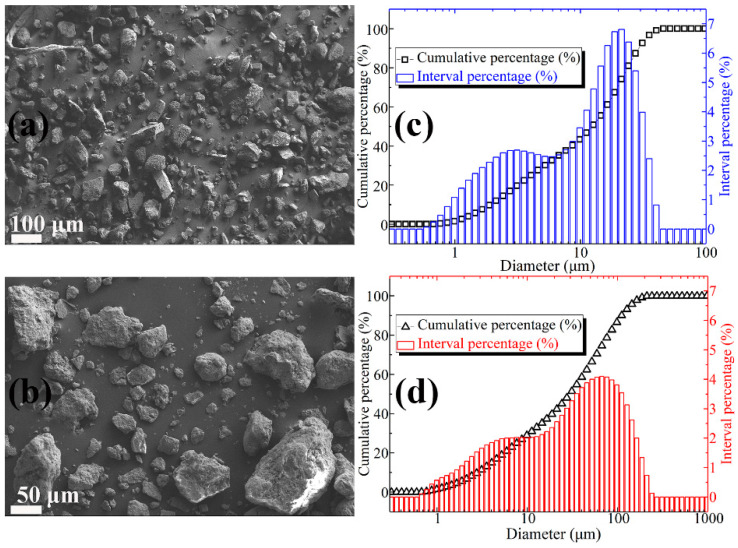
(**a**) SEM image of MgO particles; (**b**) SEM image of FD particles; (**c**) particle size distribution of MgO powder; (**d**) particle size distribution of FD powder.

Figure 1a shows the SEM image of lightly burned magnesium oxide. It can be observed from Figure 1a that the MgO particles are not uniform in size, irregular in shape, and sharp in edges and corners. From the particle size distribution curve (Figure 1c), it can be concluded that the particle size of magnesia is between 0.5 micron and 40 micron. Among them, the particle size of 2 micron and 20 micron is the most. The SEM micrographs of FD are shown in Figure 1b. The dust particles are irregular in shape and uneven in size, but the surface is relatively smooth. Moreover, it can be concluded from Figure 1d that the content of dust particles in the range of 1–5 micron, 5–10 micron, 10–50 micron, and 50–100 micron is 17.49%, 14.08%, 26.74%, and 27.58%, respectively, and the total content is 85.89%. The main chemical compositions of the lightly burned magnesium oxide powder and the FD expressed by the percentage of oxide weight are listed in Table 2 and Table 3, respectively. It can be seen that the content of magnesium oxide in lightly burned magnesia is as high as 92.08%. The main impurity in light burned magnesia is silica, and the content is 4.82%. As a comparison, the composition of FD is relatively complex. It consists of 46.55% silica, 21.74% alumina, 10.33% magnesium oxide, 7.85% calcium oxide and other oxides (Na_2_O, SO_3_, K_2_O, TiO_2_, MnO, and Fe_2_O_3_). The sum of the contents of the above four oxides is as high as 86.47%.

### 3.2. XRD Analysis

**Figure 2:**
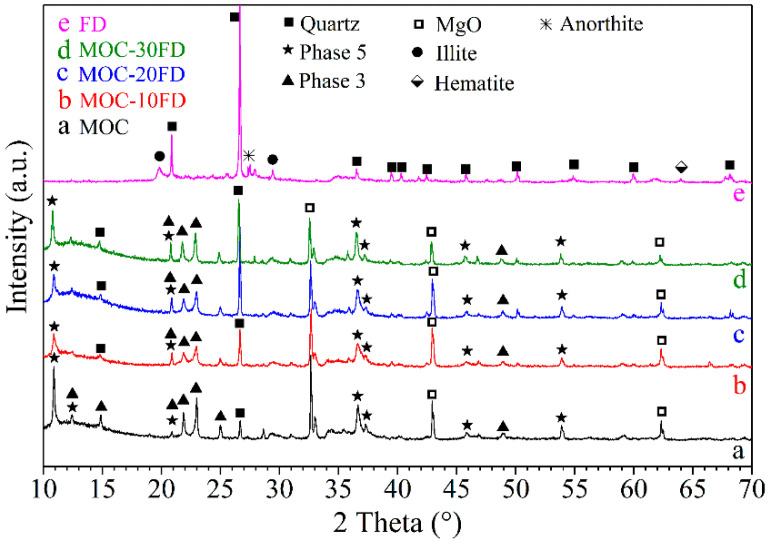
XRD patterns of different samples: a. pure MOC; b. FD/MOC composites containing 10 wt.% of FD; c. FD/MOC composites containing 20 wt.% of FD; d. FD/MOC composites containing 30 wt.% of FD; e. FD powders.

Figure 2 shows the XRD spectra of FD powders, pure MOC paste, and MOC paste with different FD content. It can be observed that Phase 5 [5Mg(OH)_2_·MgCl_2_·8H_2_O], Phase 3 [3Mg(OH)_2_·MgCl_2_·8H_2_O], periclase, and quartz exist in the pure MOC paste. The formation of Phase 5 and Phase 3 could be described by the following chemical reactions:

(2) $$\mathrm{Phase}\;5:\;5MgO+MgCl_{2}+13H_{2}O\to 5Mg{\left(OH\right)}_{2}\cdot MgCl_{2}\cdot 8H_{2}O$$

(3) $$\mathrm{Phase}\;3:\;3MgO+MgCl_{2}+11H_{2}O\to 3Mg{\left(OH\right)}_{2}\cdot MgCl_{2}\cdot 8H_{2}O$$

Periclase can be attributed to the incomplete reaction of magnesia in light calcined magnesia, while quartz can be assigned to the silica impurity in the raw material. Additionally, the main crystalline phase in the FD is quartz, with a small amount of anorthite and hematite. The XRD spectrum of the MOC/FD composite is almost the same as that of the pure MOC (see Figure 2b–d). However, with the increase of the content of FD in the composite, the XRD peak intensity of quartz phase in the composite increases correspondingly, while the XRD peak intensity of periclase decreases. The above results are in good agreement with the content of each component in the MOC/FD composite.

### 3.3. SEM Analysis

**Figure 3:**
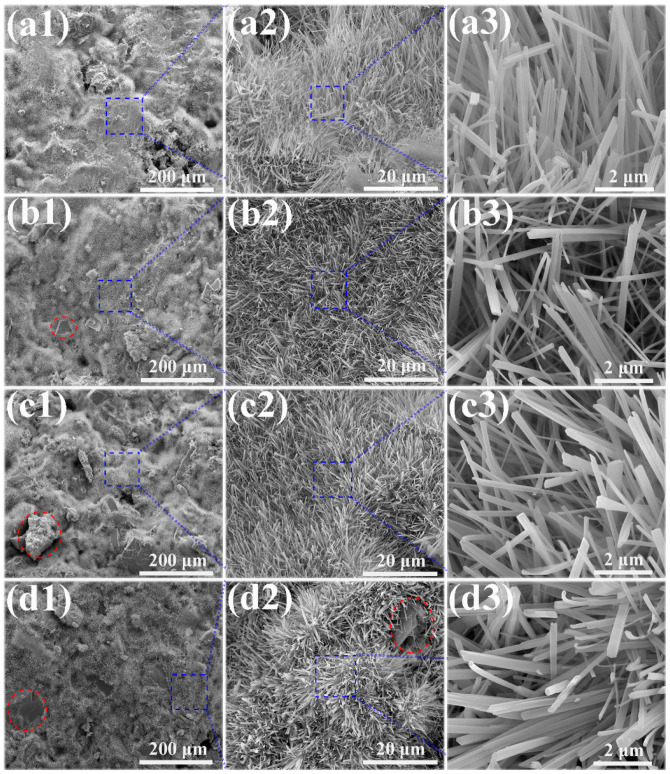
SEM morphologies of the fractured surfaces of different samples. (**a1**), (**a2**) and (**a3**) pure MOC; (**b1**), (**b2**) and (**b3**) MOC-10FD; (**c1**), (**c2**) and (**c3**) MOC-20FD; (**d1**), (**d2**) and (**d3**) MOC-30FD.

**Figure 4:**
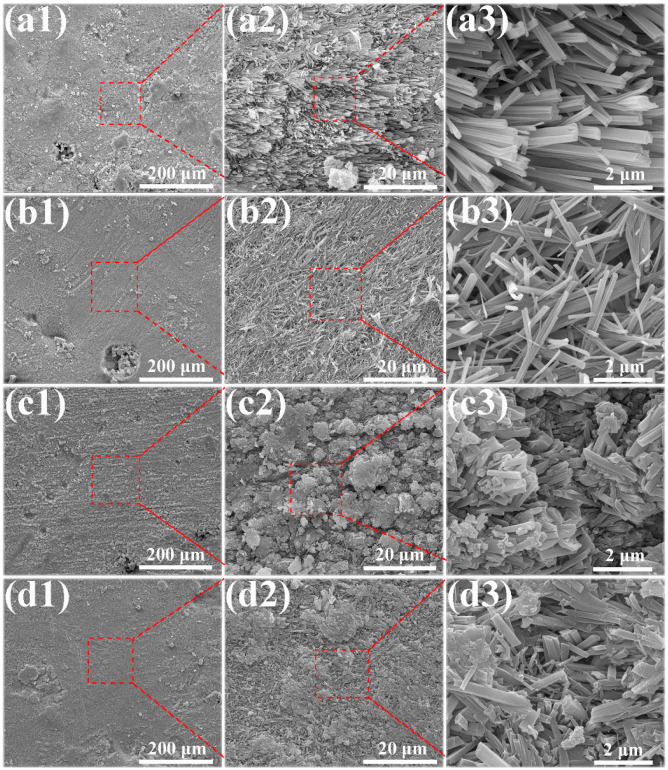
SEM morphologies of the as-prepared surfaces of different samples. (**a1**), (**a2**) and (**a3**) pure MOC; (**b1**), (**b2**) and (**b3**) MOC-10FD; (**c1**), (**c2**) and (**c3**) MOC-20FD; (**d1**), (**d2**) and (**d3**) MOC-30FD.

The microstructures of the MOC/FD composites with different FD content (i.e., 0%, 10%, 20% and 30%) are depicted in Figure 3. A large number of needle-like crystals exist that can be observed on the fracture surfaces of the MOC/FD composites (see Figure 3(a2–d2,a3–d3)), which is the unique morphology of the MOC. The diameter of most needle crystals is about 0.2 µm, and there is little difference between them (see Figure 3(a3–d3)). The length of the needle crystals cannot be accurately estimated from the figures (Figure 3(a3–d3)), but it is much longer than 2 microns. FD particles are tightly wrapped by needle-like crystals, as shown in Figure 3(b1–d1,d2). In short, the structure of the FD/MOC composite is very dense, thus ensuring the large mechanical strength of the composite. Surface FE-SEM images of the FD/MOC composites with different FD content are shown in Figure 4. One can see that the surfaces of all kinds of FD/MOC composites are also very dense (see Figure 4(a1–d1,a2–d2)). The presence of FD particles is hardly observed on the composite surface. At high magnification, it can be observed that the needle-like crystals of MOC are staggered and lie flat on the surface. When the FD content is 20% and 30%, the length of needle crystals on the surface of the FD/MOC composite is significantly shorter than that of pure MOC and FD/MOC composite with low FD content Figure 4(a3–d3)).

**Figure 5:**
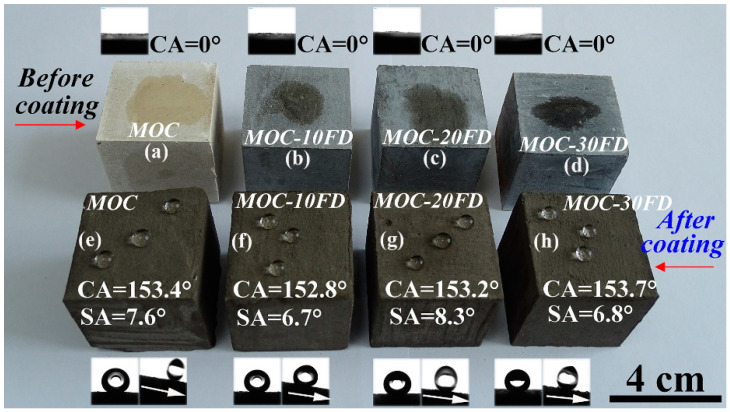
Photograph of the wetting behavior of water droplets on various FD/MOC composite surfaces. (**a**)–(**d**) before coating treatment; (**e**)–(**h**) after coating treatment. The inset show the CA and/or SA measurement.

**Figure 6:**
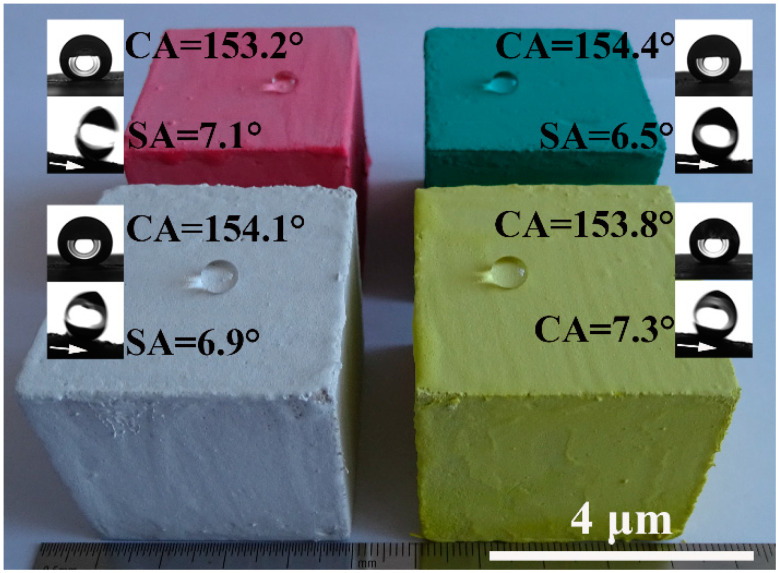
Superhydrophobic PC coatings with various colors, the substrates used are FC/MOC composites containing 30 wt.% FD.

Pure MOC is yellow (see Figure 5a). After incorporated with FD, the color of the composite becomes gray, which is similar to the appearance of hardened PC paste. The most prominent disadvantage of MOC is the lack of water resistance. One can see that the FD/MOC composites are superhydrophilic (CA = 0°) because water droplet wets the surfaces easily (Figure 5a–d). After PC coating treatment, the FD/MOC composites possess superhydrophobicity [CA > 150° and SA < 10°]. Ball-like water droplet sit on the surfaces of PC coating and they can roll off the surfaces easily (Figure 5e–h). The color of the coating is dark gray, which makes it look dull, but the color of the coating can be adjusted and beautified with pigments (Figure 6).

**Figure 7:**
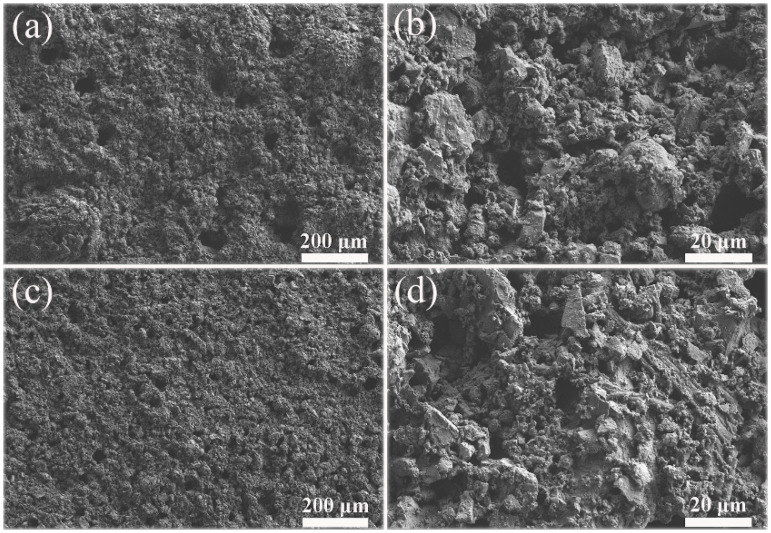
FE-SEM images of two kinds of PC coatings: (**a**) and (**b**) PC coatings without using hydrophobic emulsion; (**c**) and (**d**) PC coating containing hydrophobic emulsion.

**Figure 8:**
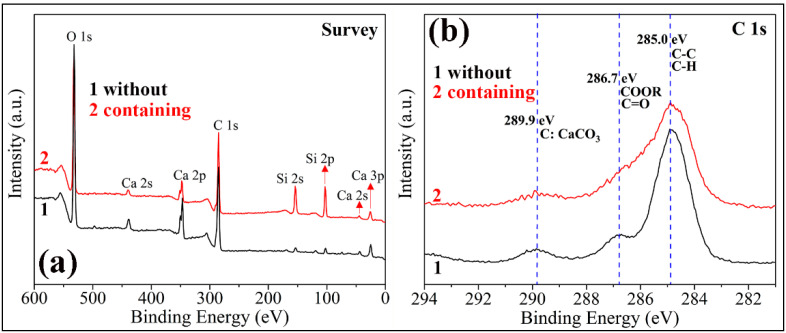
XPS spectra of two kinds of PC coatings (without using hydrophobic emulsion and containing hydrophobic emulsion): (**a**) survey spectra; (**b**) high resolution spectra in C 1s region.

The FE-SEM images of two kinds of PC coatings were measured to compare the influence of hydrophobic emulsion on the surface wettabilities (please see Figure 7). The surface of the cement coating without hydrophobic emulsion consists of many micron-level holes and protrusions, which is a rough surface (Figure 7a,b). In contrast, the surface morphology of superhydrophobic PC coating containing hydrophobic emulsion has no obvious change (Figure 7c,d). The chemical composition of the two coating surfaces was analyzed using XPS (Figure 8). One can see that the element Ca, C, and Si were detected by XPS for the PC coating without using hydrophobic emulsion. Calcium and silicon are derived from the hydration products of PC, namely calcium silicate gel (C-S-H) and calcium hydroxide (CH). Carbon is derived from calcium carbonate formed after carbonation of CH. Compared with the emulsion-free coating, the elements detected in the XPS spectrum of the emulsion-containing coating are the same as the former, but the silicon element intensity is significantly reduced (Figure 8a). The C 1s spectra of the two kinds of coatings were depicted in Figure 8b. The XPS spectrum of the emulsion-free coating shows three distinct peaks, which are located at 289.9 eV, 286.7 eV and 285.0 eV, respectively. The XPS peak at 289.9 eV can be assigned to the carbonized product calcium carbonate [24]. The XPS peak at 286.7 eV was assigned to the adsorption of polar organic compounds on PC coating surface [25]. The XPS peak at 285.0 eV is attributed to the adsorption of non-polar organic compounds on the surface of PC coating [26,27]. The hydrophobic emulsion contains elements such as silicon, carbon and oxygen and they were detected by XPS survey spectrum (see Figure 8a). The XPS C1s spectrum of the emulsion-containing coating contains a distinct peak (285.0 eV) corresponding to the non-polar carbon in silane/siloxane emulsion (CH_3_ and/or CH_2_). The two peaks located at 289.9 eV and 286.7 eV are very weak or even diminished, which can be explained by the fact that the buffer function of silane/siloxane emulsion. Silane in the coating delayed the carbonization of hydration products and prevented the coating from adsorbing polar organic compounds from the air. The SEM and XPS test results show that the rough structure of the cement hydration product naturally formed on the coating surface combined with the low surface energy provided by the hydrophobic emulsion gives the emulsion-containing PC coating surface superhydrophobicity.

**Figure 9:**
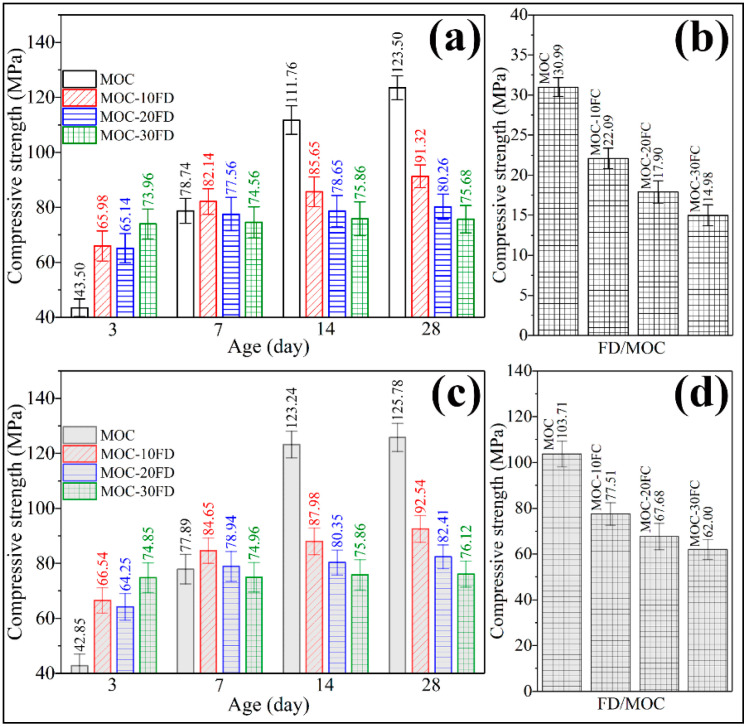
Compressive strength measurement of the FD/MOC composites. (**a**) FD/MOC composites without coating before soaking in water; (**b**) FD/MOC composites without coating after soaking in water; (**c**) FD/MOC composites containing coating before soaking in water; (**d**) FD/MOC composites containing coating after soaking in water.

Figure 9a shows the compressive strength of the coating-free FD/MOC composites at different curing ages before soaking in water. Compared with the FD/MOC composites, pure MOC has lower strength in the early stage (3 days, 43.50 MPa) and higher strength in the later stage (14 days, 111.76 MPa, and 28 days, 123.50 MPa). Therefore, the addition of FD is beneficial to the development of the early strength of MOC, but has an adverse effect on the late strength of MOC. For the long-term use of FD/MOC composite, the more FD added, the lower the strength of the FD/MOC composite. The relative strength of the FD/MOC composites with 30% FD decreased by 38.7% compared with that of pure MOC. However, due to the high strength of MOC matrix, the absolute strength of the FD/MOC composite is still as high as 75.68 MPa. The strength of the FD/MOC composite is much higher than that of the corresponding Portland cement-based composites, which can meet the application needs of most construction engineering fields. It was found that the superhydrophobic coating has little effect on the mechanical properties of the FC/MOC composites by comparing Figure 9a with Figure 9c. However, the superhydrophobic coating has great influence on the water resistance of the composite. As can be seen from Figure 9b, the FD/MOC composites without coating exhibit compressive strength lower than 6.0 MPa after soaking in water for 7 days. The water resistance of the FD/MOC composite modified by superhydrophobic coating has been greatly improved. Even after soaking in water, the compressive strength of the FD/MOC composite containing superhydrophobic coating is still above 60 MPa. The softening coefficients (SC) of the FD/MOC composites before and after superhydrophobic coating modification were summarized in Table 4. It can be seen from Table 4 that the SC of the composite without coating is less than 0.26, while that of the composite containing coating is greater than 0.81. Therefore, the method proposed in the present work of using superhydrophobic cement coating to improve the water resistance of MOC and MOC-based composites is effective.

## 4. Conclusions

In the present work, MOC was used to recycle FD. FD is beneficial to improve the early strength of FD/MOC composites, but not to the long-term strength development.

The FD has no hydration activity in the MOC, and only plays the role of physical filling. When the FD content is 30 wt.%, the compressive strength of the FD/MOC composite is greater than 75 MPa, much higher than the corresponding PC-based composites. The softening coefficient of the D/MOC composite is very low, less than 0.26. A superhydrophobic coating was prepared on the surface of FD/MOC composite by using silane/siloxane aqueous emulsion as modifier, PC as film forming agent and water as solvent. The surface CA of the PC-based coating is greater than 150° and SA is less than 10°. The water resistance of the FD/MOC composite modified by the PC-based superhydrophobic coating is obviously improved, and its softening coefficient is more than 0.81. The FD/MOC Composites can be used to produce building blocks by using solid wastes on a large scale. The superhydrophobic coating on the FD/MOC Composites’ surface ensures the water resistance and self-cleaning of the building blocks.

## Figures and Tables

**Table 1:** The formulation of the FD/MOC composites with different FD concentration.

Specimen	Mass (g)				Molar Ratio	
	MgO	MgCl_2_·6H_2_O	FD	H_2_O	H_2_O/MgCl_2_	MgO/MgCl_2_
Pure MOC	426.7	214.0	0	1080.0	10.0	9.0
MOC-10FD	384.0	214.0	42.7	1080.0	10.0	8.1
MOC-20FD	341.4	214.0	85.3	1080.0	10.0	7.2
MOC-30FD	298.7	214.0	128.0	1080.0	10.0	6.3

**Table 2:** The oxide composition of MgO powders measured by XRF.

Compound	MgO	Al_2_O_3_	SiO_2_	CaO	MnO	Fe_2_O_3_
Wt.%	92.08	1.05	4.82	1.75	0.02	0.28

**Table 3:** The oxide composition of FD measured by XRF.

Compound	Na_2_O	MgO	Al_2_O_3_	SiO_2_	SO_3_	K_2_O	CaO	TiO_2_	MnO	Fe_2_O_3_
Wt.%	3.51	10.33	21.74	46.55	0.90	2.36	7.85	1.02	0.05	5.69

**Table 4:** Softening coefficient measurement for the FD/MOC composites before and after superhydrophobic coating treatment.

Material	FD Content	Softening Coefficient	
		Without Coating	Containing Coating
Pure MOC	0	0.251	0.843
FD/MOC composite	10	0.242	0.838
FD/MOC composite	20	0.223	0.821
FD/MOC composite	30	0.198	0.815

## Abbreviations

FD	foundry waste
MOC	magnesium oxychloride cement
PC	Portland cement
CA	contact angle
SA	sliding angle
